# A Cost Effectiveness and Capacity Analysis for the Introduction of Universal Rotavirus Vaccination in Kenya: Comparison between Rotarix and RotaTeq Vaccines

**DOI:** 10.1371/journal.pone.0047511

**Published:** 2012-10-24

**Authors:** Albert Jan van Hoek, Mwanajuma Ngama, Amina Ismail, Jane Chuma, Samuel Cheburet, David Mutonga, Tatu Kamau, D. James Nokes

**Affiliations:** 1 Immunisation, Hepatitis and Blood Safety Department, Health Protection Agency, London, United Kingdom; 2 Kenya Medical Research Institute, Centre for Geographic Medicine Research-Coast, Kilifi, Kenya; 3 Division of Disease Surveillance and Response, Ministry of Public Health and Sanitation, Nairobi, Kenya; 4 Health Information System, Ministry of Health, Nairobi, Kenya; 5 Division of Vaccines & Immunization, Ministry of Public Health and Sanitation, Nairobi, Kenya; 6 School of Life Sciences, University of Warwick, Coventry, United Kingdom; Groningen Research Institute of Pharmacy, United States of America

## Abstract

**Background:**

Diarrhoea is an important cause of death in the developing world, and rotavirus is the single most important cause of diarrhoea associated mortality. Two vaccines (Rotarix and RotaTeq) are available to prevent rotavirus disease. This analysis was undertaken to aid the decision in Kenya as to which vaccine to choose when introducing rotavirus vaccination.

**Methods:**

Cost-effectiveness modelling, using national and sentinel surveillance data, and an impact assessment on the cold chain.

**Results:**

The median estimated incidence of rotavirus disease in Kenya was 3015 outpatient visits, 279 hospitalisations and 65 deaths per 100,000 children under five years of age per year. Cumulated over the first five years of life vaccination was predicted to prevent 34% of the outpatient visits, 31% of the hospitalizations and 42% of the deaths. The estimated prevented costs accumulated over five years totalled US$1,782,761 (direct and indirect costs) with an associated 48,585 DALYs. From a societal perspective Rotarix had a cost-effectiveness ratio of US$142 per DALY (US$5 for the full course of two doses) and RotaTeq US$288 per DALY ($10.5 for the full course of three doses). RotaTeq will have a bigger impact on the cold chain compared to Rotarix.

**Conclusion:**

Vaccination against rotavirus disease is cost-effective for Kenya irrespective of the vaccine. Of the two vaccines Rotarix was the preferred choice due to a better cost-effectiveness ratio, the presence of a vaccine vial monitor, the requirement of fewer doses and less storage space, and proven thermo-stability.

## Introduction

Rotavirus is the major cause of severe dehydrating diarrhoea in infants and young children worldwide. Two vaccines have recently undergone efficacy trials in sub-Saharan Africa and the results are viewed as supportive of the introduction of universal infant vaccination [1]. The GAVI Alliance (GAVI) has pledged support for vaccine introduction in eligible countries in the region, and Kenya falls into such a category [2]. However, there are many factors, social, economic, epidemiologic and programmatic which need to be weighed up before introducing a new vaccine into the schedule of the Expanded Programme on Immunization (EPI) in Kenya. This study aims to address some of these issues in collaboration with the Ministry of Public Health and Sanitation (MoPHS), Kenya, to provide suitable data and analytical outcomes to support a decision to introduce rotavirus vaccine. A classical cost-effectiveness analysis is presented, using a deterministic model framework supported wherever possible by in-country data relating to vaccine delivery, uptake, fixed, recurrent, opportunity costs, incidence and efficacy. The key question addressed in this work is which of the two vaccines would be the best option?

## Methods

### Setting and Sources of Data

Kenya is a developing country in East Africa with an estimated 41 million inhabitants and a population growth rate of 2.46% a year. As an indicator of the strength of EPI implementation, the uptake of the third dose of the pentavalent DTP/HepB/Hib vaccine by the end of the first year of life is 86%. The health system in Kenya is categorized into 6 levels. First level are community health workers, second level are dispensaries and clinics, third level are health centres, maternity and nursing homes, fourth level are district and private hospitals, fifth level are provincial hospitals and sixth level are referral (national) hospitals. Incidence of disease in the outpatient (ie dispensary and health centres; level 2 and 3) and the hospital (principally District hospitals; level 4, 5 & 6) settings were investigated together with mortality rates in the community and hospital. In the sections below the sources of data and estimates of key epidemiological and economic variables and their possible ranges (Table 1), and the details of the cost-effectiveness model are set out. Also discussed is the estimated impact of each of the vaccines on the cold chain.

**Table 1:** Characteristics of Rotarix and RotaTeq vaccines for the prevention of rotavirus associated diarrhoea.

	Rotarix	Source	RotaTeq	Source
Manufacture	Glaxo Smith Kline	[11]	Merck	[12]
Doses	2 doses	[11]	3 doses	[12]
Vaccine virus	G1P(8)	[11]	G1,G2,G3,G4,P1A(8)	[12]
Concentration	10^6^ CCID­	[11]	2.2×10^6^ G1, 2.8×10^6^ G2, 2.2×10^6^ G3, 2.0×10^6^ G4, 2.3×10^6^ P1A(8)	[12]
Cross potection	G1, G3, G4, G9	[11]	G1,G2,G3,G4	[12]
Administration	Oral	[11]	Oral	[12]
Timing of doses	6–24 weeks	[11]	6–32 weeks	[12]
Interval between doses	Minimal 4 weeks	[11]	4 to 10 weeks	[12]
Store temp vial	2–8 degrees celcius	[11]	2–8 degrees celcius	[12]
Use within	24 hours	[12]	As soon as possible	[12]
Package size vial	17.1cm3 per dose (full course: 34.2cm3)	[38]	46.3cm3 per dose (full course: 138.9cm3)	[38]
Vaccine Vial Monitor technology	Yes	[38]	No	[38]
Price	$2.5 per dose, $5 full course	[25]	$3.5 per dose, $10.5 full course	[26]
Shelf life	24 months	[11]	24 months	[12]
Adverse events	Low	[11]	Low	[12]
Thermostability	Proven	[37]	Unproven	

### The Burden of Disease and National Health Care Use

The incidence of diarrhoeal disease resulting in out-patient medical attention was determined from two sources. The first source was the Kenya Demographic and Health Survey (KDHS) [3], a household-based population and health survey over the 8 provinces of Kenya. The mothers of 5,481 children born within the preceding 5 years were asked if their child experienced diarrhoea for which medical attention was sought in the two weeks before the interview (definition not specified). Of those children 442 (8%) had diarrhoea in these two weeks. Using this data and assuming a duration of 5 days an annual incidence of diarrhoea, I_KDHS_, was estimated [4] where I_KDHS_  =  (Number of children with diarrhoea/Number of children sampled) * (period of recall for diarrhoea/(period of recall + duration of illness) * recall periods per year * 100,000. Hence I_KDHS_  =  ((442/5,481)*(14/(14+5))*26)*100,000 = 154,834 diarrhoea cases per 100,000 children under five years.

As a second source data was obtained from the Health Information System (HIS), a national system obtaining summary data from the registers of all health care facilities. The percentage of double counting is estimated at 1% (personal communication S. Cheburet, Ministry of Health). In 2010 there were 971,746 records of outpatient visits due to diarrhoea among children under 5 years of age, amounting to 7% of the total health visit records (13,895,226 visits). Given the number of children under five years of age to be 5,939,306 in 2009 [5], and correcting for the proportion of health facilities reporting (84% in 2010), this yields an incidence, I_HIS_, of 19,478 (95% CI: 19,439–19,517, see Table 2) per 100,000 children under 5 years of age per year. There is an 8-fold difference between the two estimates I_KDHS_ and I_HIS_. For the base case scenario the conservative estimate was used, and the higher incidence was included in the sensitivity analysis.

**Table 2:** Overview assumptions cost effectiveness model in the base case.

Parameter	Point estimate	Distribution	Source
*Disease incidence*			
Diarrhoea requiring outpatient health care	19,478 per 100,000	Poisson (95% CI: 19,439–19,517)	HIS
Percentage diarrhoea in the outpatient caused by rotavirus	15.5% (24/155)	Binomial (95% CI: 10.2%–22.2%)	WHO surveillance Health care clinic THC & NHC Year 2010
Diarrhoea requiring hospitalisation	1,595 per 100,000 (978 per 100,000 after adjustment)	Poisson (95% CI: 1,516–1,669)	Tate et al. JID 2009, Nokes et al. Plos Med 2008
Adjustment for national estimate	61.3%	None	KDHS
Percentage diarrhoea hospitalisation cause by rotavirus	28.6% (303/1059)	Binomial (95% CI: 26.0%–31.4%)	WHO surveillance; Embu provincial hospital, Kilifi district hospital and Siaya district hospital; year 2010
Mortality in the hospital	2.0% (6/303)	Binomial (CI 95%: 0.9%–4.3%)	WHO surveillance; Embu provincial hospital, Kilifi district hospital and Siaya district hospital; year 2010
*Mortality*			
Under 5 mortality	74 per 1000 new born		[3]
Percentage mortality caused by diarrhoea	14.5% (428/2954)	Binomial (95% CI: 13.2%–15.8%)	[10]
Percentage diarrhoea mortality caused by rotavirus	28.6% (303/1059)	Binomial (95% CI: 26.0%–31.4%)	WHO surveillance; Embu provincial hospital, Kilifi district hospital and Siaya district hospital; year 2010
Percentage diarrhoea mortality caused by rotavirus – sensitivity analysis	42.9% (982/2289)	Binomial (95% CI: 40.8%–45.2%)	WHO surveillance Kenyatta National Hospital, year 2007, 2008, 2009 & 2010
*Vaccine efficacy*			
Outpatient cases	57%	None	Assumed
Hospitalised cases	57%	None	[8]
Mortality	78%	None	[8]
*Costs disease – heatlh care related*			
Outpatient	$23.7 (2132 Ksh)		[18]
Hospital	$29.7 (2673 Ksh).		[18]
*Costs disease – societal related*			
Outpatient	$3.2 (286Ksh)		[21]
Hospital	$19.9 (1795 Ksh)		[21]
*Disability Adjusted Life Year* 1			
DALY weight diarrhoeal disease; Outpatient	0.086	None	[22]
DALY weight diarrhoeal disease; Hospitalization	0.119	None	[22]
Life expectancy	59		[3]
*Cost vaccination*			
Administration costs	$1.4		[21]
Vaccination coverage (final coverage 1 year)			
1^st^ dose	95.8%		[3]
2^nd^ dose	93.1%		[3]
3^rd^ dose	86.4%		[3]
Discount rate	3%		
Costs	2011 Dollars	1 dollar = 90KES	

1 DALYs were estimated using the formula developed by Murray et.al assuming a beta of 0.04 and a constant of 0.1685.

The incidence of diarrhoea requiring hospitalisation in children under 5 years of age was based on hospital surveillance in the District of Kilifi. In 2002–2004 there were 1,706 cases of diarrhoea admissions to Kilifi District Hospital (KDH) among 107,224 person-years of observation leading to an incidence of 1,591 per 100,000 person-years (95% CI; 1,516–1,668) [6]. However the KDHS recorded diarrhoeal disease as more prevalent on the coast than elsewhere in the country. Hence, the National incidence of hospitalisations due to diarrhoea was obtained by scaling the KDH estimate by the ratio of the National incidence over the Coastal Province incidence observed in the KDHS, ie 61.3% (252,731 per 100,000/154,834 per 100,000). This results in an estimate of National incidence of hospitalised diarrhoea of 975 per 100,000 children under five years of age per year (95% CI; 929–1,022).

The proportion of diarrhoea caused by rotavirus (among children aged under 5y) is monitored via the World Health Organisation in-country sentinel surveillance system. Surveillance is conducted at 4 sites in Kenya; Kenyatta National hospital in central province, Embu/Mau district hospital in Eastern province, Kilifi district hospital in coast province and Siaya district hospital in Nyanza province and two health care centres; Ting’wani and Njenjra also in Nyanza province. The sentinel surveillance system therefore covers different geographical areas and different levels of care. Cases of bloody diarrhoea, cases with duration of symptoms over 7 days, and cases of hospital acquired gastroenteritis were excluded from the analysis, as those were considered not to be virus related or reflecting disease in the community. Using data on the duration of diarrhoea (days), diarrhoea episodes, duration of vomiting (days), vomiting episodes, fever, dehydration status and treatment it was possible to score disease severity on the Vesikari scale [7], see also the supporting material Table S1. In line with the published clinical trials a score of 11 or above was considered severe [8], [9]. To circumvent seasonal variation effects only data from full surveillance years were included.

In the two health care facilities of Siaya District the overall prevalence of rotavirus in diarrhoea cases was 15.5% (24 of 155, 95% CI: 10.2%–22.2%) in 2010, in the District and Provincial hospitals the prevalence of rotavirus was 28.6% (303/1059, 95% CI: 26.0%–31.4%) for the same period, and in Kenyatta National hospital the prevalence was 42.9% (982/2289, 95% CI: 40.8%–45.2%) measured over 2007 to 2010. Based on the Vesikari score disease treated in Kenyatta National Hospital, which provides end of line care, was remarkably more severe (mean Vesikari score of 18.1) compared to disease treated in the other health care facilities (maximum mean Vesikari score 13.4 or lower), see also the supporting material Figure S3.

The under five incidence of rotavirus diarrhoea was estimated by multiplication of the outpatient or hospital incidence for diarrhoeal disease by the 15.5% disease caused by rotavirus in the health care clinic (outpatient) or 28.6% hospital setting and subsequently redistributed over the first 60 months of life by the observed age distribution of diarrhoea in the health care clinic or hospital. The age distribution was smoothed by Friedman’s super smoother function in R (version 2.11.1) to reduce the influence of month-by-month fluctuations (see the supporting material Figure S1 and Figure S2 for the data points and the smooth fit).

### Mortality Due to Diarrhoeal Disease and Rotavirus

Under 5 mortality in Kenya, taken from the KDHS of 2008–9, was 74 per 1,000 live births in 2009 [3], with the mortality risk per month of age decreasing from 31 per 1000 in the first month to 1.9 per month in the age group 1–11 months and 0.46 per 1000 live births per month in year 1 to 4. In a survey of mortality caused by gastroenteritis/dehydration in 2954 children aged less than 5 years in Western Kenya between 1^st^ of May 2002 and 31st December 2005 [10], the main cause of death was related to diarrhoea in 14.5% (n = 428) children (95% CI: 13.2%–15.8%). Mortality due to rotavirus was calculated from the under five mortality multiplied by the proportion caused by diarrhoea and the proportion caused by rotavirus as observed in the hospital. The total number of deaths was attributed to a month of age using the smoothed age distribution of hospitalised cases as described above. In the sensitivity analysis the mortality caused by rotavirus was investigated based on the observed percentages of death caused by rotavirus observed at the different levels of care covered by the WHO surveillance, see Table 2.

### Vaccine Efficacy

Two vaccines are currently available to prevent diarrhoeal disease due to rotavirus; a monovalent human rotavirus vaccine of genotype G1P(8) (delivered in 2 doses at weeks 6 and 10) known under the trade name Rotarix™ and produced by GlaxoSmithKline (GSK) [11], and a bovine backbone vaccine consisting of genotypes G1, G2, G3, G4 and P1A(8) (3 doses; at week 6, 10 and 14) called RotaTeq® produced by Merck&Co.INC. [12]. Both vaccines consist of live attenuated virus with proven cross protection against at least some other genotypes [11], [12]. The vaccines can be administered concurrently with DTP/HepB/Hib, pneumococcal conjugate vaccines and oral polio vaccination (OPV). Further characteristics of these vaccines are listed for comparison in Table 1.

Both vaccines showed similar vaccine efficacy among African populations [8], [9] in the first year of life, and only the RotaTeq surveillance continued into the second year of life. In the trial of Rotarix in South Africa and Malawi the observed efficacy (per protocol) against hospitalisation was 57.5% (95% CI: 7.2%–80.8%) in the first year of life. The vaccine efficacy was more profound against severe diarrhoea (≥11 Vesikari scale) with 76.9% (95% CI: 56.0%–88.4%). A trial of RotaTeq in three countries (Ghana, Kenya and Mali) had a combined efficacy of 64.2% (40.2–79.4) in the first year of life. In Kenya alone (Nyanza province) efficacy was 83.4% (95% CI: 25.5%–98.2%) for the prevention of severe disease (Per protocol, ≥11 Vesikari scale) in the first year of follow up. However, in the second year of follow-up in Kenya there was a higher disease burden in the vaccine arm compared to the placebo, leading to a negative vaccine efficacy.

Various factors complicate the interpretation of the observed disease reduction; the observed efficacy is dependent on disease severity, with greater impact against more severe disease; the host population characteristics influence the impact [13], as impoverished or malnourished population tend to respond less well; circulating genotypes during the trial, where lower response is expected when the circulating strains are not vaccine related [8]. Differences between research protocols and presentation of results, complicates comparison between trials from the two vaccines [14]. In the analysis that follows we therefore assumed an identical efficacy for both vaccines, as was done before [15]. The vaccine efficacy against hospitalized disease (Rotarix in Malawi and South Africa, 57.5%) was applied for the prevention of outpatient and hospitalised disease. The vaccine efficacy against severe disease (Rotarix in Malawi and South Africa, 76.9%) was applied in the prevention of mortality, as it was assumed that mortality occurred in patients with severe disease. Although RotaTeq has three doses we assumed that full protection was achieved after the second dose. The efficacy between the first dose and second dose was assumed at 50% of the efficacy after the full course. As the protective effect after the first year of age was not surveyed in the clinical trials of Rotarix and was negative in the trial of RotaTeq in Kenya, vaccine efficacy was set to 0 after the first year of life as the baseline. In a sensitivity analysis efficacy in second and subsequent years was set to 50% and 100% of its first year estimate.

**Figure 1:**
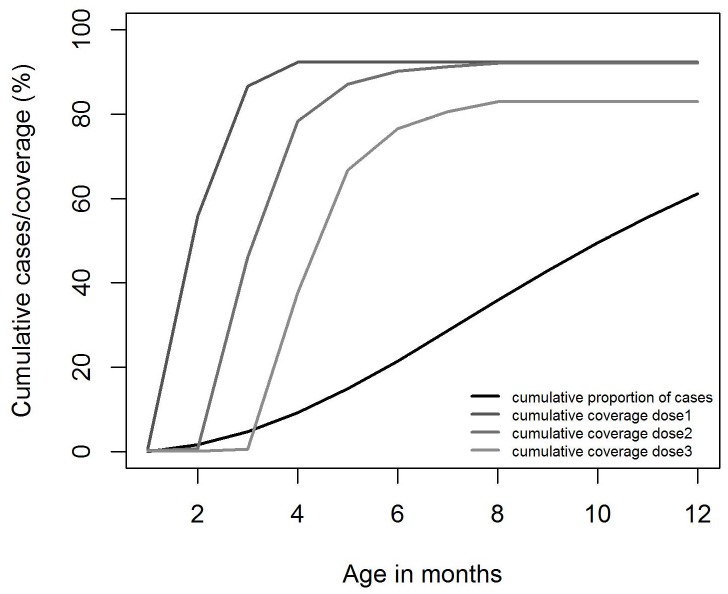
The cumulative coverage of one, two and three doses of vaccine (lines of decreasing greyness) and the cumulative proportion of mortality (dark grey) by month of age over the first year of life.

A previous rotavirus vaccine (Rotashield) was associated with increased incidence of intussusception [16] and children who received the vaccine later in life appeared to be under greatest risk of this adverse outcome [17]. It is therefore advised that the first dose of any rotavirus vaccine should be given before week 15, and the last dose before week 32 [1]. Due to this constraint the exact timing of vaccination was incorporated in the analysis. Rotavirus vaccine was modelled as given at the same time as DTP/HepB/Hib. Use is made of the actual timing of administration of DTP/HebB/Hib as a proxy using data from the Pneumococcal Conjugate Vaccine Impact study in Kilifi, Coastal Province. Data were available form 1^st^ January 2009 to 15^th^ July 2011 for a total of 36,144 doses. Of the recipients of a first dose of DTP/HebB/Hib within the first 12 months of life 96.4% were vaccinated by 4 months of age. Of those receiving a 2^nd^ and 3^rd^ dose within their first year, 98.9% and 96.0% respectively were vaccinated within the first 8 months of life. Overall vaccination coverage was based on KDHS data, with a reported coverage at 12 months of age of 95.8% for dose 1, 93.1% for dose 2 and 86.4% for dose 3. Final coverage was a product of the coverage at 12 month and the cumulative percentage in each month receiving the vaccine. See Figure 1 for the overall coverage, per dose, by month of age.

### Costs Due to Disease

Costs were estimated for the outpatient visit and hospital admissions. Direct medical cost were based on an in-depth survey of the actual costs (based on institutional documentation and audited accounts) in the different levels of the Kenyan health care system in 2006/2007 performed by Flessa et al. [18]. Cost estimations were available specific for diarrhoea diagnosis and by the three different providers of care; Government, Non Governmental Organisations (NGO)/Faith Based Organisations (FBO) and private institutions. Direct costs include medication, supplies and staff and indirect costs related to running the facility. Costs were deflated up to 2011 by the Consumer Price Index of Kenya [19]. Patients were distributed over the level of provider of care based on the observed health care seeking behaviour for children less than 5 years of age as recorded in the Household Health Expenditure and Utilisation Survey; a cluster randomized household survey among 8,453 households from all regions of Kenya executed in 2007 [20]. The estimated unit cost for an outpatient (dispensary) visit was US$23.7 (2132 KSh) and hospitalisation was US$29.7 (2673 KSh).

Direct and indirect medical costs were estimated by Tate et al. [21] in 2007 among a sample of 94 hospitalized diarrhoea cases and 124 diarrhoea cases in the health care clinic. The costs include transportation, medication and diagnostic tests and lost income. To prevent double counting, facility fees were deducted from the published totals. The estimated total costs amounted to US$3.2 (286KSh) for an outpatient visit and US$19.9 (1795 KSh) for a visit to the hospital. Total costs for an outpatient visit including direct and indirect costs were therefore US$26.9 (2419KSh) for an outpatient visit and US$49.6 (4468KSh) for a visit to the hospital.

### Disability Adjusted Life Year

The prevention of the burden of disease was expressed in Disability Adjusted Life Years (DALY). The assumed DALY weight was 0.086 for outpatient and 0.119 for hospitalised diarrheal disease [22] and 1 for death. DALY weights were discounted and age weighted as defined by Murray et al. [23]. Disease was assumed to last 5 days.

### Impact of Introduction of the Vaccine into the EPI

The impact of the introduction of rotavirus vaccination on the storage capacity at various levels of the cold chain (national, regional, district, facility) was evaluated with the Cold Chain Equipment Manager (CCEM). This is a Microsoft Access based software package developed by PATH [24] and used by the Ministry of Public Health and Sanitation in Kenya. Within the software package there is a function to measure the impact of new vaccination programmes. In Kenya vaccines are distributed from the national vaccine stores in Nairobi to 8 regions, and those regions supply 138 different district locations who further distribute vaccines to 5158 vaccination facilities. The 5158 facilities comprise dispensaries (3046), health centres (887), hospitals (470), private medical clinics (642) and nursing homes (113). Most are managed by the government (62%), with 20% managed privately, 13% by FBOs and 5% by NGOs. Three scenarios were investigated; the capacity under the current vaccination schedule; introduction of Rotarix (2 doses, volume 17.1 cm2 per dose) into the current schedule; introduction of RotaTeq (3 doses, volume 46.3 cm2 per dose). Both vaccines had a targeted coverage of 85% and wastage of 5%. Storage capacities were obtained from a national inventory of the total cold chain executed in February 2011 by the ministry. We assumed that each dispensary was supplied on a monthly basis. Capacity shortage was calculated based on the local storage capacity and vaccine demand per location (volume demand/volume capacity), as estimated based on the vaccine coverage and population in the catchment area of each location. Missing population data was imputed based on national averages and constrained such that it did not exceed the regional population size as estimated in the 2009 census. Storage capacity was considered problematic when the capacity shortage exceeded 10%.

### Cost Effectiveness Model

A monthly cohort was followed for the first 5 years of life. Monthly incidence was assigned based on the under 5 incidence and the observed age distribution. The analysis was performed from the societal perspective. Results are presented as number of cases and the cost per DALY. Future costs and benefits were discounted by 3% per annum. The used vaccine price was US$2.5 per dose for Rotarix [25] and US$3.5 for RotaTeq [26] based on formal announcements by the manufactures, and an administration cost of US$1.4 per dose was added [21]. Probabilistic sensitivity analysis was performed using applicable distributions for each parameter as presented in Table 2; from each distribution 1000 samples were drawn by Latin hypercube sampling. All analyses were performed in R 2.11.1.

There are three cost effectiveness thresholds used by the WHO; (i) highly cost effective, defined as lower than the GDP per capita (corrected for purchasing-power-parity), (ii) cost-effective, defined as one to three times GDP, and (iii) not-cost effective, defined as more than three times the GDP. The GDP for Kenya in 2010 was US$1600 resulting in the thresholds of US$1600, US$1600-US$4800 and >US$4800 for the three categories, respectively.

Scenario analysis was performed on the key assumptions; these are mortality, cost assumptions, vaccine efficacy, waning of vaccine efficacy and incidence of outpatient disease.

## Results

### Burden of Disease

The median estimated incidence of rotavirus disease in Kenya was 3015 outpatient visits, 279 hospitalisations and 65 deaths per 100,000 children under five per year. In a cohort of 1,200,000 this accumulated to a median total number of 170,228 outpatient visits, 15,744 hospitalisations and 3679 deaths. Of the deaths an estimated 304 (8.3%) occurred in the hospital. The cases in the health care system had an attached cost of US$3,932,237 for outpatients and US$454,886 for in-patients; the societal costs were estimated to be US$530,935 among outpatients and US$304,789 for hospitalised patients. The total DALYs lost were 183 DALY among outpatients, 23 DALY among hospitalized patients and 116,565 DALY due to mortality.

### Impact of the Vaccine

**Figure 2:**
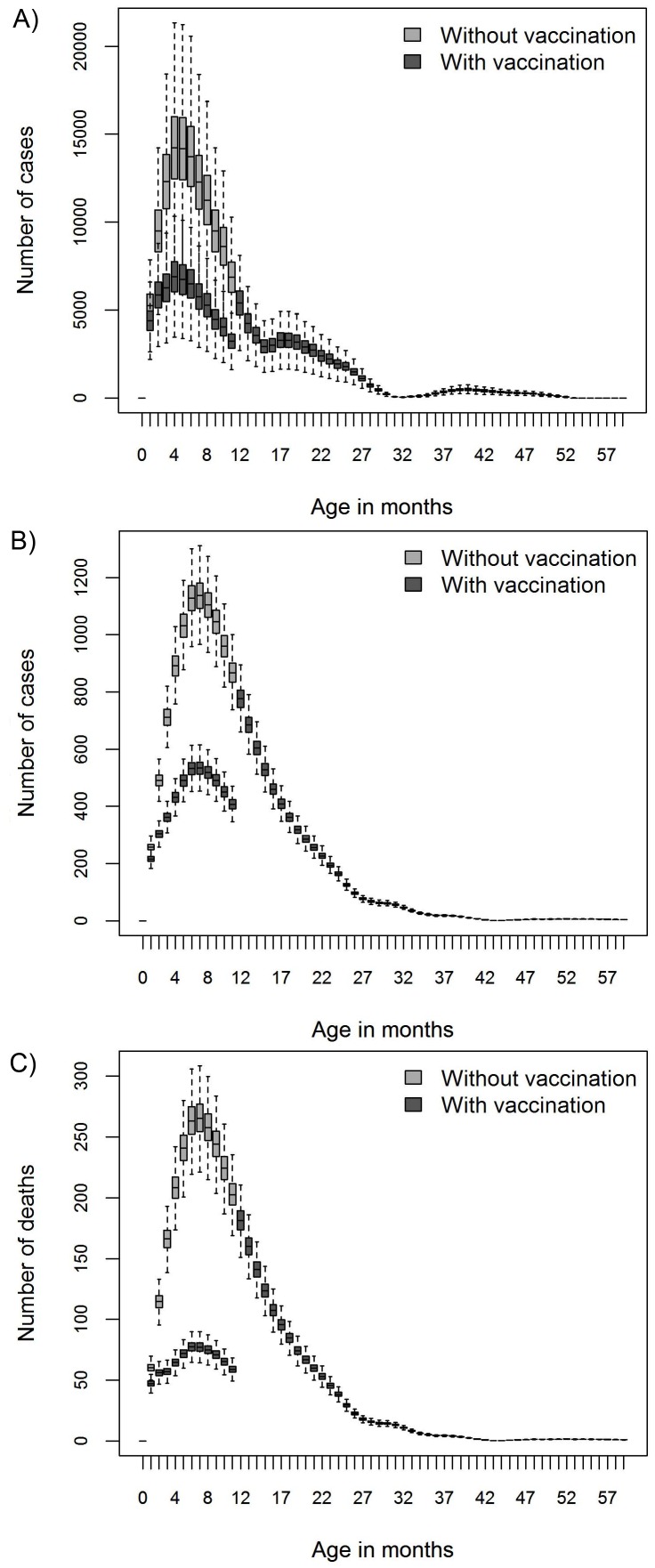
The health care use associated with rotavirus in Kenya, by month of age, observed before (light grey bars) and predicted after (dark grey bars) introduction of a rotavirus vaccine. The panels represent the number of outpatient clinic visits (panel a), hospital admissions (b) and deaths (c). The figures are representative for both Rotarix and RotaTeq as an identical efficacy was assumed.

Cumulated over the five years included in the study vaccination was predicted to prevent 34% of the outpatient visits, 31% of the hospitalizations and 41.5% of the deaths, associated with rotavirus; or it prevented 58,262 outpatient visits, 4,866 hospitalisations, and 1,527 deaths, due to rotavirus. Incidence associated with rotavirus was reduced to 1,984 outpatient visits, 192 hospitalizations and 38 deaths per 100,000 children aged under 5y, Figure 2 and Table 3. The model estimated that, over the five year period, vaccination would prevent costs associated with rotavirus amounting to US$1,503,370 to the health care system and US$279,392 to the society, yielding a total cost prevention of US$1,782,761. The corresponding number of prevented DALYs was estimated to be 48,551 from a total of 116,771 resulting in a reduction of 42%.

**Table 3:** Overview of the number of cases, discounted costs and DALYs, programme costs and Cost/DALY for the situation without vaccination and with vaccination and the prevented number of cases and costs.

	Without vaccination	With Rotarix	With RotaTeq	Prevented (difference Rotarix and rotaTeq)
Outpatient visits	170,228	111,966	111,966	58,262
*Incidence per 100,000*	*3,015*	*1,983*	*1,983*	*1,032*
Hospital visits	15,744	10,858	10,858	4,886
*Incidence per 100,000*	*279*	*192*	*192*	*87*
Deaths	3,679	2,152	2,152	1,527
*Incidence per 100,000*	*65*	*38*	*38*	*27*
Deaths in the hospital	304	178	178	126
Outpatient costs: health care	$3,932,237	$2,572,286	$2,572,286	$1,359,950
Outpatient costs: society	$530,935	$347,313	$347,313	$183,622
Hospital costs	$454,886	$312,210	$312,210	$142,676
Hospital costs	$304,789	$209,191	$209,191	$95,597
DALY outpatient	183	120	120	63
DALY hospital	23	16	16	7
DALY mortality	116,565	68,084	68,084	48,481
Doses	–	2,213,247	3,208,264	(995,017)
Program cost	–	$8,631,662	$15,720,492	(7,088,830)
Cost/DALY: health care	–	$147	$293	($146)
Cost/DALY: society	–	$142	$288	($147)

Presented number is the median outcome of 1000 latin hypercube samples.

### Cost Effectiveness

**Figure 3:**
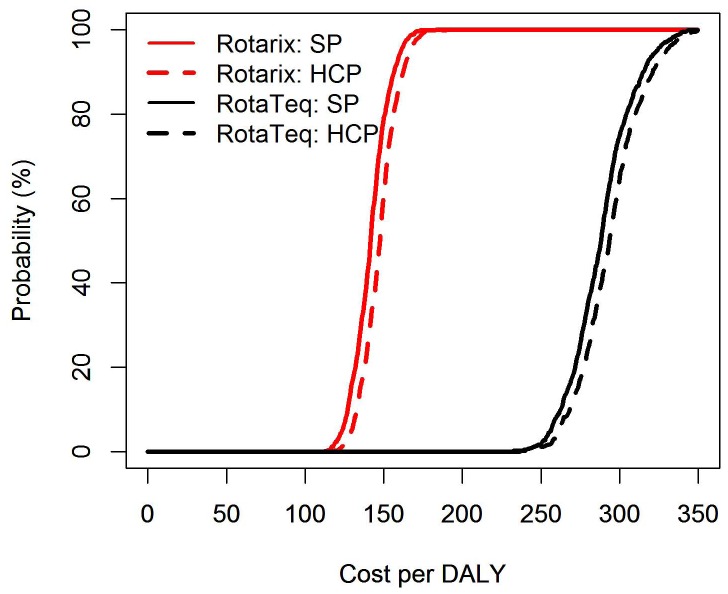
Cost-effectiveness acceptability curve for Rotarix (red) and RotaTeq (black) following vaccine introduction in Kenya modelled over a five year period. The Societal Perspective (SP) is the solid line, the Health Care Perspective is marked by the dashed line.

To achieve this vaccine impact a total of 2.2 million doses were needed in the case of Rotarix and 3.2 million for RotaTeq. The costs for a childhood vaccination programme were US$8.6 million for Rotarix and US$15.7 million for RotaTeq. Due to the difference in costs of the programme the estimated cost effectiveness from a societal perspective was an average US$142 per DALY for Rotarix (US$147/DALY from a health care perspective) and US$288 per DALY for RotaTeq (US$294/DALY from a health care payer’s perspective). See Figure 3 for the cost-effectiveness acceptability curves. Both vaccines can be marked highly cost effective under WHO criteria.

### Sensitivity Analysis

Several scenarios were applied to investigate the sensitivity of the model to different assumptions, and results are presented in Table 4. The first scenario does only include observed mortality in the hospital, excluding non-observed but estimated community mortality; this increased the ICER to US$2,289/DALY for Rotarix and US$4,461/DALY for RotaTeq, reducing the vaccine from highly cost effective, to cost effective. As mortality is so influential two related scenarios were investigated. Assuming a similar proportion of diarrhoea deaths caused by rotavirus as observed in the outpatient clinic (15.5%) led to a base line incidence of 35 deaths per 100,000, and resulted in a cost/DALY of US$271/DALY for Rotarix and US$551/DALY for RotaTeq. Then, setting the observed proportion of mortality to that observed in Kenyatta national hospital (42%), resulted in an incidence of 98 death per 100,000 children under five, and yielded a ICER of US$94/DALY for Rotarix and US$191 for RotaTeq. In both scenarios the cost effectiveness remained “very cost effective” under the WHO criteria. Given the threshold of $1600 per DALY saved and the average DALY lost per death (31.7 DALY) you can obtain the minimum number of death among the 5,646,450 children under 5 in the model as we observed a reduction of 41.5% in the overall rotavirus mortality measured over the total five years (see above). When the savings of costs are ignored the minimal incidence of rotavirus mortality has to be 7.9 per 100,000 (0,4 per 1000 life births) for Rotarix and 14.8 per 100,000 (0,7 per 1000 life births) for RotaTeq.

**Table 4:** Scenario sensitivity analysis.

Scenario	Rotarix	RotaTeq
Base case	$142	$288
No discounting	$139	$283
Ignore community mortality	$1,688	$3,414
Ignore costs	$178	$342
High incidence outpatient (23,974 per 100,000)	Cost saving	$66
Protection after first year 50% of the vaccine efficacy	$100	$210
Protection after first year 100% of the vaccine efficacy	$75	$163
% diarrhoea death due to Rotavirus 15.5% (as in the outpatient); incidence 35 per 100,000	$262	$535
% diarrhoea deaths due to Rotavirus 42,5% (as in Kenyatta national hospital); incidence 98 per 100,000	$94	$191
10% lower vaccine efficacy for all outcomes (47,5%/66,9%)	$170	$338
20% lower vaccine efficacy for all outcomes (37,5%/56,9%)	$209	$406

Outcome in the cost per DALY for various scenarios. For each scenario the median Cost per DALY are shown (societal perspective).

In the second scenario, the costs of outpatients and hospitalisation were excluded from the calculation to investigate the influence of these costs on the outcome, generating an ICER of US$178 per DALY for Rotarix and US$325 per DALY for RotaTeq. The third scenario investigated the assumption on the waning of vaccine-induced immunity. When a 50% reduction of the vaccine efficacy was applied beyond the first year of life (instead of a 100% reduction as in the base case) the ICER dropped to US$100 per DALY for Rotarix and US$210/DALY for RotaTeq. The final scenario investigated the possible under estimation of outpatient visits, applying the estimates for outpatient visits based on the KDHS, increasing the outpatients visits to 23,976 per 100,000 children under five, which pushed the use of Rotarix into cost saving and RotaTeq to an ICER of US$66 per DALY.

### Capacity

The current capacity in the cold chain was estimated to be almost sufficient on a national and regional level. There were problems in 40 of the 138 (29%) districts and 617 of the 5158 (12%) local locations, see Table 5. Introduction of Rotarix was determined to cause pressure on the cold chain at district level, with an increase of 27 districts with a shortage of 10% or higher. At local facility level an estimated extra 102 locations were estimated to end up in a capacity deficit. Introduction of RotaTeq caused capacity problems at all levels in the cold chain. Due to the added volume of the RotaTeq vaccine both the national storage capacity as well as the capacity in one of the regions would be insufficient. Compared to the current programme an extra 76 districts would experience capacity deficits as well as 411 facilities, resulting in deficits at 1146 of the total 5305 (22%) vaccine storage sites.

**Table 5:** Number of vaccine storage locations with an estimated shortage of more than 10% as estimated in CCEM for the current vaccination schedule, introduction of Rotarix (2 doses, volume 17.1 cm2 per dose), or introduction of RotaTeq (3 doses, volume 46.3 cm2 per dose).

Level	Number vaccine storage sites	Current programme	Introduction Rotarix	Introduction RotaTeq
National	1	0 (0%)	0 (0%)	1 (100%)
Regional	8	0 (0%)	0 (0%)	1 (13%)
District	138	40 (29%)	67 (49%)	116 (84%)
Facility	5158	617 (12%)	719 (14%)	1028 (20%)
Total	5305	657 (12%)	786 (15%)	1146 (22%)

Assumed was a coverage 85% and wastage 5%.

## Discussion

The intention is to introduce a rotavirus vaccine into the vaccination programme of Kenya in the near future. Based on efficacy data in sub-Saharan Africa this introduction is expected to result in a significant reduction of the disease burden caused by this virus. Two vaccines are available with similar efficacy for protection against severe rotavirus in the first year of life. Applying vaccine prices per dose of US$3.5 for RotaTeq or US$2.5 for Rotarix in the analysis both vaccines can be considered cost effective using the WHO threshold. Furthermore, the conclusion of ‘cost effective’ for both vaccines is robust. Firstly, in our analysis herd protection was not included although this has been reported [27], inclusion of this will improve the cost effectiveness. Secondly, even if only the death and disease burden as registered in the health care system are considered (rather than that assumed to arise in the community not recorded directly by the health system) the verdict remains ‘cost effective’. Notwithstanding the above, in all scenarios Rotarix will be more cost-effective, and create less strain on capacity than RotaTeq due to the fewer vaccine doses (lower cost; less storage volume).

The major factor influencing the cost effectiveness estimation is rotavirus-associated mortality, most of which is assumed to arise outside the health care system. The uncertainties originate in the estimates of (i) the mortality rate in children aged less than five years, (ii) the proportion of mortality due to diarrhoea and (iii) the proportion of diarrhoea caused by rotavirus, where the last in the list is the most uncertain. The under five mortality is based on a household survey of Kenyan mothers who gave birth in the preceding five years with a sample size of only 3973 and thus of dubious precision [3]. However, since mortality tends to be under-reported, the uncertainty will in all likelihood lead to under-estimated disease burden. The estimated value of 74 per 1000 live births is lower in comparison to similar surveys in the recent history [3]. In Ethiopia, a neighbouring country, 23% of the mortality was found to be caused by diarrhoea in a recent study [28]; the estimated percentage based on a meta-analysis was 19% in Africa [29], suggesting our estimate (14.5%) is on the conservative side. The percentage diarrhoea mortality caused by rotavirus was set at the value observed from the district and provincial hospital data (29%). The proportion of disease caused by rotavirus was higher in Kenyatta National Hospital (42%), where concomitantly the average Vesikari score was substantially higher compared to lower level hospitals. If, as this suggests, the percentage of rotavirus is higher among the more severe diarrhoea cases, then the mortality due to rotavirus may be under-estimated in our study using the lower prevalence of rotavirus in diarrhoea cases. Nonetheless, the overall estimated incidence of rotavirus mortality (65 per 100,000) is comparable with the 68 per 100,000 that was estimated by Tate et.al [21] (which uses the same method, but different data).

In addition, the analysis is strongly influenced by estimates of incidence of rotavirus associated disease burden in the hospital and outpatient facilities. The average incidence of hospitalisation of 279 per 100,000 is high relative to the 132 per 100,000 estimated by Tate et al. [21] for Siaya District of Western Kenya. The true incidence of hospitalisation will differ by region, this due to true differences in disease incidence and access to health care [6]. Accordingly, the incidence estimate for diarrhoeal hospital admissions in our study was scaled to the national estimates of diarrhoeal admission incidence measured in the KDHS. Uncertainty is also associated with the incidence of rotavirus related outpatient visits. The estimated incidence of outpatient visits of 3016 per 100,000 is low compared to the 21,800 clinic visits per 100,000 children under five estimated by Tate et al [21], but high compared to the 932 per 100,000 estimated in India [30]. We based our incidence on the recorded outpatient visits which are nationally counted. The alternative, to use disease incidence 8-fold higher based on KDHS questions to mothers about diarrhoea in their infants (154,834 cases per 100,000), was investigated in the sensitivity analysis leading to more favourable cost effectiveness.

There are several issues relating to uncertainty surrounding the estimates of vaccine efficacy for rotavirus vaccines that might have a bearing the reported results. Firstly oral vaccines are less efficacious in developing countries [8], [9], [13], [14], [31], for ill-defined reasons. Secondly the vaccine does not protect against all serotypes; there is an ongoing antigenic drift and emergence of new variants due to reassortment and animal introductions [32]–[34]. The cross protection for Rotarix and RotaTeq might be different for those genotypes causing differences between the efficacy of the two vaccines. Thirdly the follow-up of vaccinated children has been short. The maximal follow-up has been 2 years after receiving the vaccine, applying the vaccine efficacy beyond this point is therefore speculative, in general the efficacy declined steeply after the first year of age. Fourthly the timing and the number of doses applied in the clinical trials differ from the schedule considered for introduction in Kenya (2 dose; at 6 and 10 weeks).

The verdict of “cost effective” depends on the applied threshold for the cost per DALY. We used the threshold of US$1,600 based on the Kenyan GDP per capita corrected for purchasing power parity, which is in line with WHO Choice definition and regional thresholds [35]. Adoption of the value used by Tate et.al [21], that is, the absolute GDP per capita (US$580; 2006), would not alter the verdict of cost effective for any of the scenarios explored in this study (Table 4) excepting where community mortality was excluded. In this case neither vaccine would be considered cost effective. There are previous estimates for the cost effectiveness of rotavirus vaccine in Kenya [21], [36]. Applying the full coarse costs we are able to compare with Tate et al. [21] who estimated a slightly higher cost per DALY for both vaccines; $168 for Rotarix (full course $7.4) and $343 for RotaTeq (full course $12.9). Comparison with Atherly et al. [36] is more difficult, as their assumption of the inclusion of administration costs and the number of doses is unclear, however all tested possibilities resulted in cost per DALY below $100 per DALY for Rotarix, and around $100 per DALY for RotaTeq. Our estimates are therefore in between previous estimates.

Finally there are other important arguments to consider in the decision between RotaTeq and Rotarix apart from cost effectiveness. These include the impact on the cold chain and the reliability of the vaccine at administration. RotaTeq has more doses and a larger volume per dose, leading to a more severe impact on the cold chain compared to Rotarix. To achieve the predicted vaccine impact it is essential that the vaccine is in good condition when it is administered. In regions with unreliable power supply the cold chain is harder to maintain. Rotarix has proven thermal stability [37] for vaccines stored at 37C for 7 days if correctly handled. Rotarix comes with a vaccine vial monitor, making it possible to see at administration if the vaccine has suffered from storage. This strongly reduces the probability that faulty vaccine is administered. In contrast, at the time of writing, RotaTeq did not have proven thermal stability and it was not available with vaccine vial monitoring technology.

## Supporting Information

Figure S1 **Fitted smooth age distribution for hospitalised cases as observed in Embu provincial hospital, Kilifi district hospital and Siaya district hospital in 2010.** The red line is diarrhoeal disease cause by rotavirus, the blue line is non-rotavirus diarrhoea. Only the red line is used in the analysis.
(TIF)

Figure S2 **Fitted smooth age distribution for patients visiting the health care clinic as observed in the health care clinic in Tingwani and Njejra (Siaya, Nyanza province) in 2010.** The red line is diarrhoeal disease cause by rotavirus, the blue line is non-rotavirus diarrhoea. Only the red line is used in the analysis.
(TIF)

Figure S3 **Distribution of Vesikari scores per WHO sentinel surveillance site.**
(TIF)

Table S1 **The used algorthim to compute the Vesikari score and the distribution of Vesikari scores per WHO sentinel surveillance site.**
(DOCX)
