# Correction: A Cost Effectiveness and Capacity Analysis for the Introduction of Universal Rotavirus Vaccination in Kenya: Comparison between Rotarix and RotaTeq Vaccines

**DOI:** 10.1371/annotation/6b9d910e-4c85-41ca-ae56-b5996e81820a

**Published:** 2012-11-21

**Authors:** Albert Jan van Hoek, Mwanajuma Ngama, Amina Ismail, Jane Chuma, Samuel Cheburet, David Mutonga, Tatu Kamau, D. James Nokes

The following information was missing from the Funding section: This work was supported by the Wellcome Trust [084633, 077092].

