# Supplementary figures and images for: A Cost Effectiveness and Capacity Analysis for the Introduction of Universal Rotavirus Vaccination in Kenya: Comparison between Rotarix and RotaTeq Vaccines
Source: PLoS One. 2012 Oct 24;7(10):e47511. doi: 10.1371/journal.pone.0047511 (PMC3480384; doi:10.1371/journal.pone.0047511)

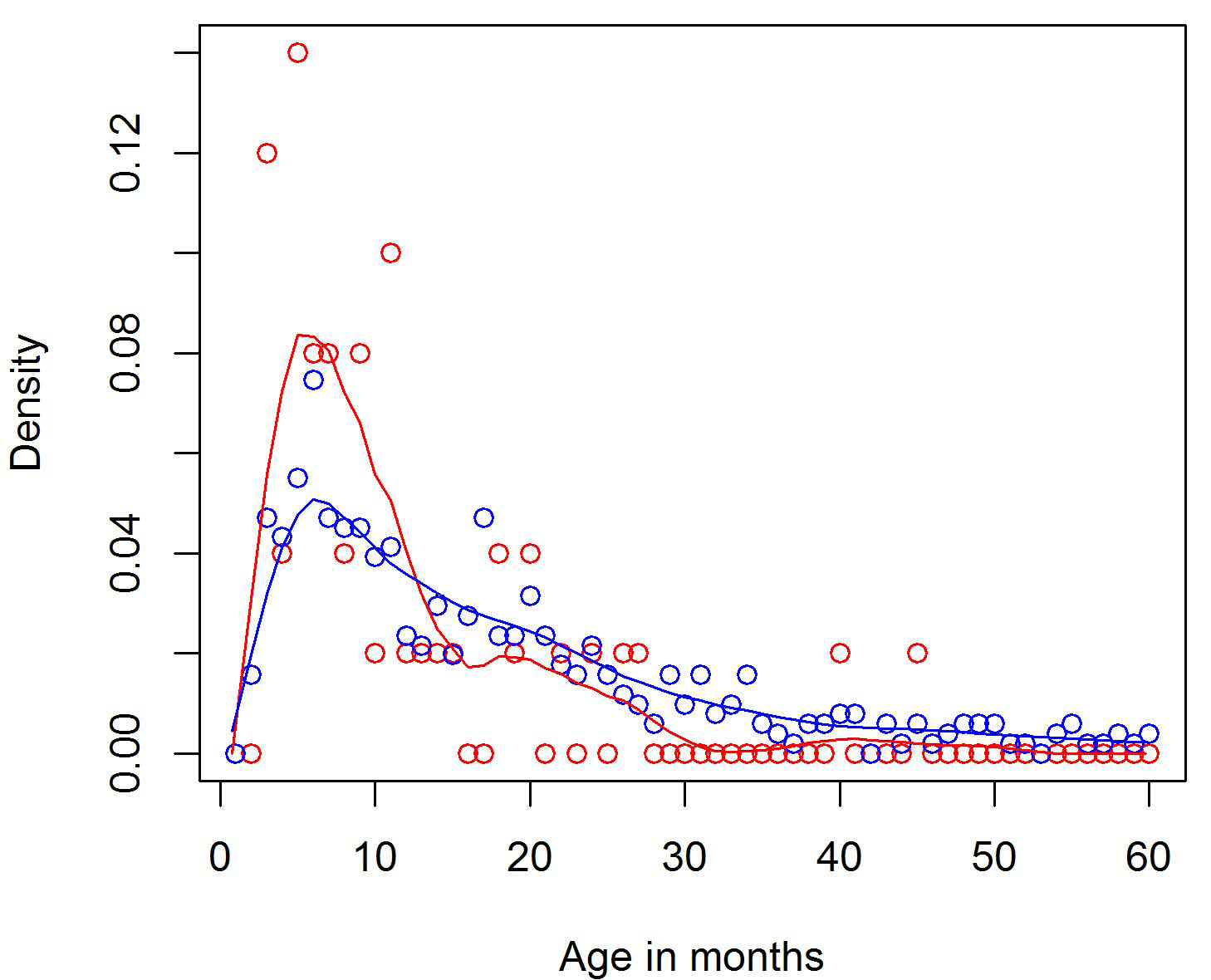

Supplement: Figure S1 — Fitted smooth age distribution for hospitalised cases as observed in Embu provincial hospital, Kilifi district hospital and Siaya district hospital in 2010. The red line is diarrhoeal disease cause by rotavirus, the blue line is non-rotavirus diarrhoea. Only the red line is used in the analysis. (TIF) [file pone.0047511.s001.tif]

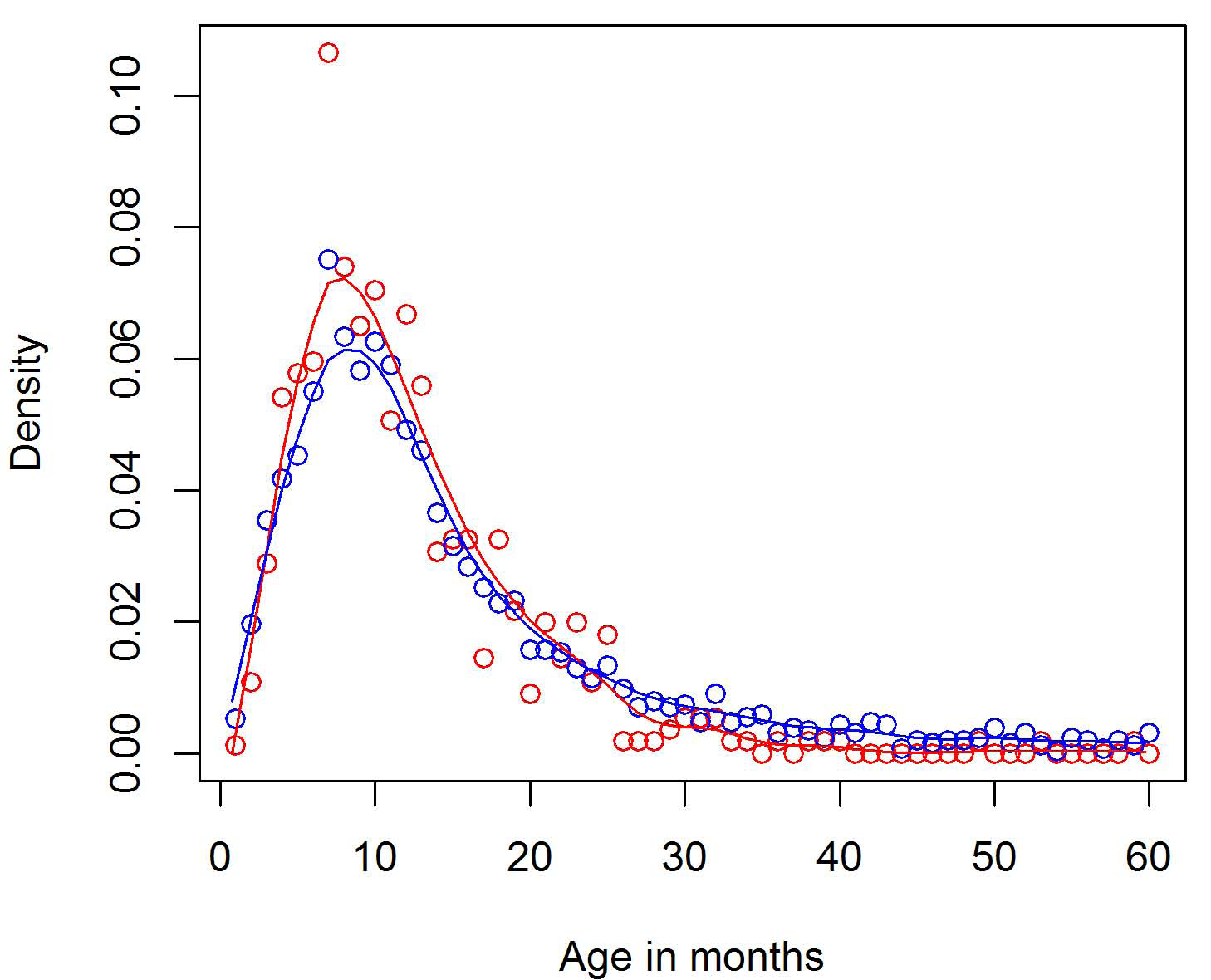

Supplement: Figure S2 — Fitted smooth age distribution for patients visiting the health care clinic as observed in the health care clinic in Tingwani and Njejra (Siaya, Nyanza province) in 2010. The red line is diarrhoeal disease cause by rotavirus, the blue line is non-rotavirus diarrhoea. Only the red line is used in the analysis. (TIF) [file pone.0047511.s002.tif]

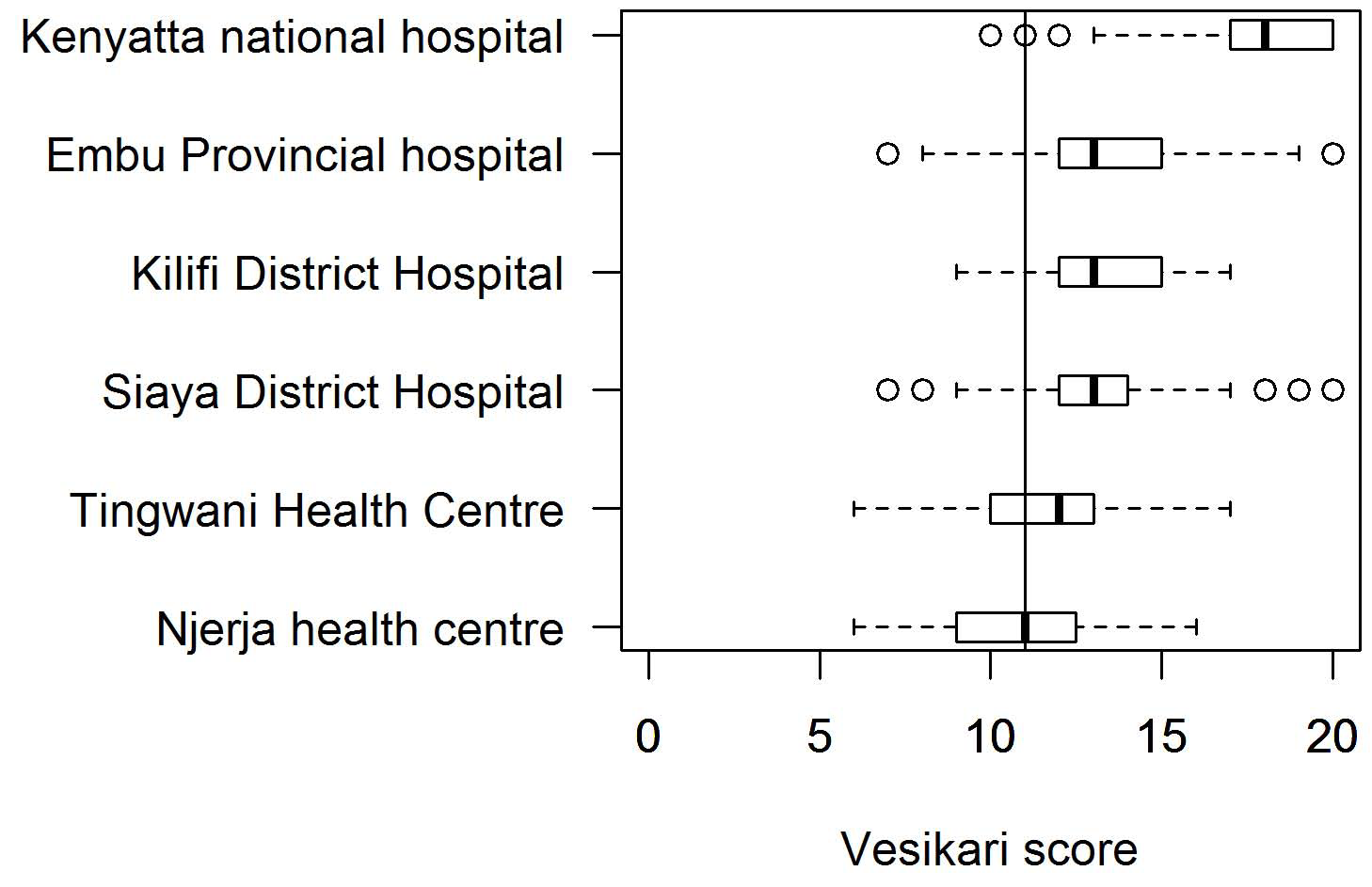

Supplement: Figure S3 — Distribution of Vesikari scores per WHO sentinel surveillance site. (TIF) [file pone.0047511.s003.tif]
