# Supplementary material for: A Cost Effectiveness and Capacity Analysis for the Introduction of Universal Rotavirus Vaccination in Kenya: Comparison between Rotarix and RotaTeq Vaccines
Source: PLoS One. 2012 Oct 24;7(10):e47511. doi: 10.1371/journal.pone.0047511 (PMC3480384; doi:10.1371/journal.pone.0047511)
Supplement: Table S1 — The used algorthim to compute the Vesikari score and the distribution of Vesikari scores per WHO sentinel surveillance site. (DOCX) [file pone.0047511.s004.docx]

The vesikari score is the sum of the following individual scores, assigned to each patient based on the collected information in the WHO surveillance:

Duration diarrhoea <5 days = 1

Duration diarrhoea = 5 days = 2

Duration diarrhoea >5 days = 3

Diarrhoea episodes < 4 = 1

Diarrhoea episodes = 4 = 2

Diarrhoea episodes > 4 = 3

Duration vomiting = 1 day = 1

Duration vomiting = 2 days = 2

Duration vomiting > 2 days = 3

Vomiting episodes = 0 = 0

Vomiting episodes = 1 = 1

Vomiting episodes = 2-4 = 2

Vomiting episodes > 4 = 3

No fever = 0

Fever 37-38.4 = 1

Fever 38.5-38.9 = 2

Fever >= 39 = 3

Dehydration “None” = 0

Dehydration “Some” = 2

Dehydration “Severe or Shock” = 3

Medical treatment = 1 (sites THC, NHC)

Hospitalised treatment = 2 (sites KNH EMB KDH and SDH)
